# *TRPM7* as a Candidate Gene for Vestibular Migraine

**DOI:** 10.3389/fneur.2020.595042

**Published:** 2020-10-23

**Authors:** Eun Hye Oh, Jin-Hong Shin, Jae Wook Cho, Seo-Young Choi, Kwang-Dong Choi, Jae-Hwan Choi

**Affiliations:** ^1^Department of Neurology, Pusan National University School of Medicine, Research Institute for Convergence of Biomedical Science and Technology, Pusan National University Yangsan Hospital, Yangsan, South Korea; ^2^Department of Neurology, Pusan National University Hospital, Pusan National University School of Medicine and Biomedical Research Institute, Busan, South Korea

**Keywords:** vestibular migraine, genetics, whole-exome sequencing, TRPM7 channel, ion homeostasis

## Abstract

**Objectives:** Vestibular migraine (VM) is a common vestibular disorder, and familial aggregation of VM with autosomal-dominant inheritance has been described, which supports a genetic background. This study aimed to describe the clinical phenotype of a family with VM, and identify a candidate gene for VM.

**Methods:** We recruited six individuals (four affected and two unaffected) from three consecutive generations of a Korean family with VM, and performed whole-exome sequencing to search for candidate genes.

**Results:** All affected individuals presented with recurrent vertigo, headache, and nausea/vomiting that fulfilled the diagnostic criteria of VM. Two individuals also experienced transient hemiparesis or dysarthria during the episodes. The symptoms were triggered by physical or emotional stress. Interictal examinations showed uni- or bi-directional horizontal gaze-evoked nystagmus in three of the individuals. They had no causative mutations in genes causing familial hemiplegic migraine or episodic ataxia. Through whole-exome sequencing from three affected individuals, we identified a nonsense mutation c.3526C>T in *TRPM7* that encodes a cation channel selective to Ca^2+^ and Mg^2+^.

**Conclusions:** Alterations in intracellular Ca^2+^ and Mg^2+^ homeostasis by *TRPM7* mutation may contribute to the development of the VM phenotype. Our result suggest that *TRPM7* is a novel candidate gene for VM.

## Introduction

Vestibular migraine (VM) is one of the most common vestibular disorders, affecting around 1% of the general population (1). It is characterized by recurrent attacks of vestibular symptoms, a current or previous history of migraine, and the existence of one or more migraine features during the vestibular episodes. Recently, VM has been included as a diagnostic category in the latest International Classification of Headache Disorders (ICHD) criteria (2).

Most cases of VM are considered sporadic, but familial aggregation of VM with autosomal-dominant inheritance has been described in several families, supporting a genetic background for the condition (3, 4). Genome-wide association studies (GWASs) have identified numerous single-nucleotide polymorphisms (SNPs) associated

with a genetic predisposition for migraine with or without aura (5, 6). Familial hemiplegic migraine (FHM), which is a subtype of migraine with aura, is caused by mutations in *CACNA1A, ATP1A2*, and *SCN1A* (7). However, no candidate gene has been validated in VM, although several genetic loci such as chromosomes 5q35, 9q13-q22, 11q, and 22q12 are known (8–11). Furthermore, previous studies have not detected pathogenic mutations in FHM or episodic ataxia (EA) genes in VM patients (12, 13).

Transient receptor potential (TRP) channels are a family of cation channels expressed mostly on the cell (14, 15). They are divided into seven subfamilies including TRPC, TRPV, TRPM, TRPA, TRPN, TRPP, and TRPML, which mediate sensory transduction such as pain, touch, hearing, and thermal sensation. In addition, TRP channels enable individual cells to respond to changes in their local environment. These ion channels have a relatively non-selective permeability to cations including Ca^2+^ and Mg^2+^, and modulate ion entry driving forces. TRP channels have been linked to neurodegenerative disorders, kidney disease, and cancers by altering intracellular ion homeostasis (15–18). Interestingly, they have been repeatedly hypothesized to contribute to migraine via the activation of meningeal nociceptors or the release of calcitonin gene-related peptide (CGRP) (19–22).

The present study investigated the clinical phenotype of a Korean family with VM showing autosomal-dominant inheritance. Here we report one novel mutation in *TRPM7* that may explain the VM phenotype in the family.

## Materials and Methods

### Subjects

**Figure 1:**
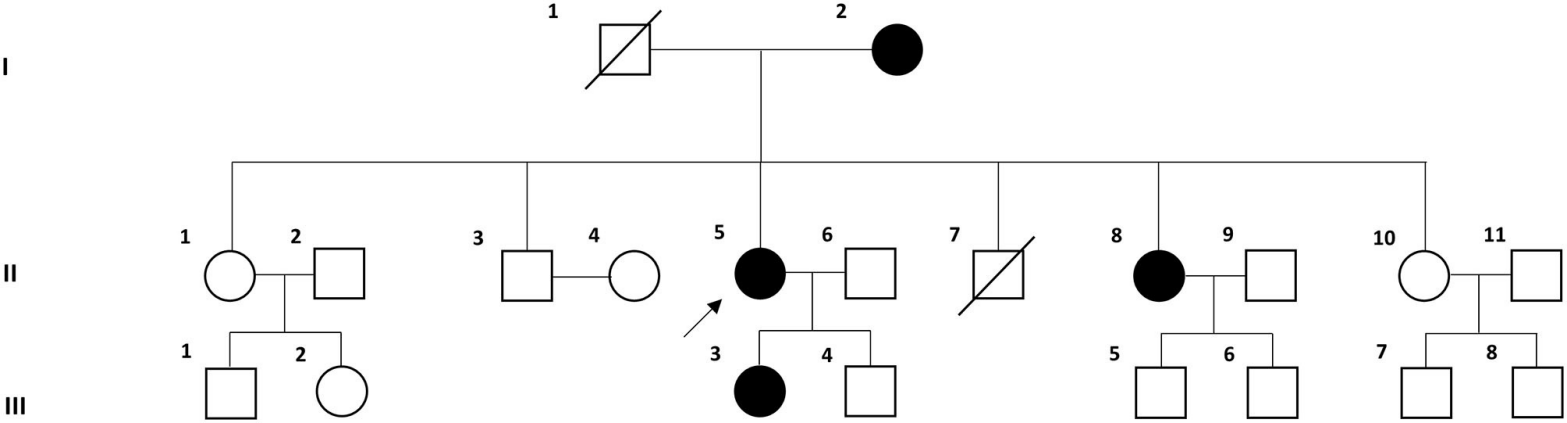
Pedigree of the Korean family with vestibular migraine. Solid symbols (squares for males and circles for females) indicate clinically affected individuals (open symbols are unaffected individuals and slashed symbols are deceased individuals). The proband is indicated by an arrow.

Six individuals from three consecutive generations of a Korean family with VM (four affected and two unaffected individuals) were recruited at the Dizziness Clinic of Pusan National University Yangsan Hospital (Figure 1). VM was diagnosed based on the criteria of the Bárány Society (1). All individuals underwent full neurological and neuro-otological examinations by the author (J-HC), and the four affected individuals received brain magnetic resonance imaging to exclude other possible causes of their symptoms.

All experiments followed the tenets of the Declaration of Helsinki, and informed consents were obtained from the participants after the nature and possible consequences of this study had been explained to them. This study was approved by the institutional review boards of Pusan National University Hospital.

### Whole-Exome Sequencing and Data Analysis

To systematically search for candidate genes, we applied trio exome sequencing to blood samples from three of the affected individuals (II-5, II-8, and III-3). Genomic DNA was extracted from the blood samples, and whole-exome sequencing was conducted using the SureSelect Focused Exome Kit (Agilent, Technologies, Santa Clara, CA, USA). Qualified genomic DNA samples were randomly fragmented by Covaris, followed by adapter ligation, purification, hybridization, and PCR. Captured libraries were analyzed using a bioanalyzer (Agilent 2100, Agilent Technologies) to estimate the quality, and they were loaded onto a sequencing system (Illumina HiSeq 2500, Theragen Etex Bio Institute, Suwon, Korea). Raw image files were processed for base-calling using HCS software (version 1.4.8) with default parameters, and the sequences of each individual were generated as 100-bp paired-end reads. Sequence reads were aligned to the human reference genome sequence (GRCh37.3, hg19) using the Burrows-Wheeler Aligner (version 0.7.12). PCR duplicate reads were marked and removed using Picard tools (version 1.92). The Genome Analysis Toolkit (version 2.3-9) was used for indel realignment and base recalibration. Variation annotation and interpretation analysis were performed using SnpEff (version 4.2).

**Figure 2:**
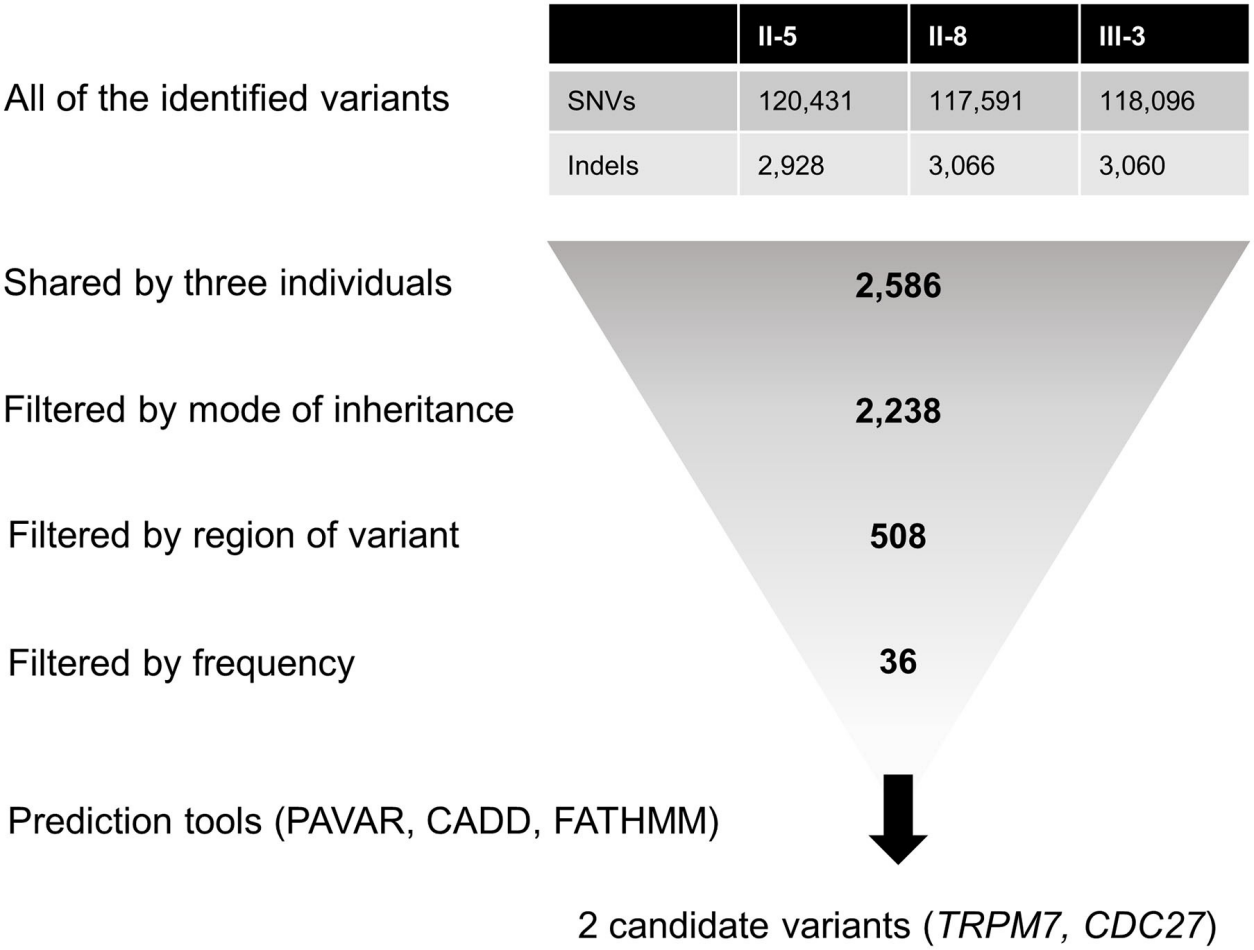
Filtering steps applied to the whole-exome sequencing data. CADD, Combined Annotation-Dependent Depletion; FATHMM, Functional Analysis Through Hidden Markov Models; Indels, insertions/deletions; PAVAR, pathogenic variants risk composite score; SNVs, single-nucleotide variants.

For candidate gene screening, we first filtered heterozygous single-nucleotide variants (SNVs) and insertions/deletions (indels) shared by all affected individuals (Figure 2). Then, variants causing non-synonymous amino acid substitutions, stop codons, indels in coding regions, and changes to splice-site sequences at exon-intron boundaries were included. Common variants with a minor allele frequency (MAF) of >0.001 in the dbSNP147, the 1,000 Genomes Project, the NHLBI GO Exome Sequencing Project (ESP), and the Genome Aggregation Database (gnomAD) were excluded. Since the frequency of variants may differ between racial groups, we further filtered rare variants with a MAF of <0.001 in the Korean Reference Genome Database (KRGDB). Candidate variants were identified and prioritized by performing functional annotation using the following tools: (1) the pathogenic variants risk composite score (PAVAR score), which is a seven-point scoring system based on annotations using SIFT (Sort Intolerant From Tolerant), PolyPhen2 (Polymorphism Phenotyping version 2), Grantham's Matrix, GERP+ (Genomic Evolutionary Rate Profiling), MutationTaster, PhastCons and PhyloP, (2) Combined Annotation-Dependent Depletion (CADD), and (3) Functional Analysis Through Hidden Markov Models (FATHMM). Variants with PAVAR score ≥5, CADD score ≥20, and FATHMM score ≤ -1.5 were considered to be potentially pathogenic (23). Finally, candidate variants were interpreted according to the standards and guidelines recommended by the American College of Medical Genetics and Genomics (ACMG) (24). The gene expression patterns were analyzed using public databases such as the Genotype-Tissue Expression (GTEx), BioGPS, and Serial Analysis of Gene Expression (SAGE), and the gene function was assessed using the HuGE Navigator, the GeneCards Human gene database, the Online Mendelian Inheritance in Man database, and PubMed. The candidate variants were validated by Sanger DNA sequencing, and were screened in another individuals within the family and 100 normal controls.

## Results

### Clinical Phenotype

The clinical characteristics of the family are summarized in Table 1. All affected individuals presented with rotatory vertigo, headache, and nausea/vomiting lasting for several hours to days. All had a history of migraine with or without visual aura, and showed one or more migraine features during the vestibular episodes. Two individuals (I-2 and II-8) experienced transient hemiparesis during the attacks, one (II-8) of whom also had dysarthria and facial dysesthesia on the same side as the hemiparesis. The symptoms were triggered by physical or emotional stress. Two individuals also had auditory symptoms such as tinnitus (II-5) and ear fullness (II-8), but air-conduction audiograms showed normal hearing functions (Supplementary Figure 1). One individual (II-8) had a past history of recurrent seizure during her childhood. Between the vestibular episodes, three individuals (I-2, II-5, and III-3) showed uni- or bi-directional horizontal gaze-evoked nystagmus. The other neurological and neuro-otological examinations were unremarkable. Three individuals who received treatments showed a good response to flunarizine (II-5 and II-8) or propranolol (III-3).

**Table 1:** Clinical characteristics of the affected family members.

**Patient no**.	**Sex/age**	**Age at onset**	**Duration**	**Ictal symptoms**	**Interictal signs**	**Migraine features**	**Additional symptoms**
I-2	F/86	50	Hours-days	Vertigo, headache, nausea/vomiting, hemiplegia	Uni-directional GEN	One sided location, pulsatile quality, moderate pain intensity, nausea	(–)
II-5	F/54	53	Hours	Vertigo, headache, nausea/vomiting	Bi-directional GEN	One sided location, pulsatile quality, moderate pain intensity, photophobia, nausea	Tinnitus
II-8	F/48	30	Hours-days	Vertigo, headache, nausea/vomiting, hemiplegia, dysarthria, facial dysesthesia	(–)	One sided location, pulsatile quality	Seizure, ear fullness
III-3	F/30	19	Hours	Vertigo, headache, nausea/vomiting	Bi-directional GEN	Visual aura, moderate or severe pain intensity, aggravation by physical activity, nausea/vomiting, photophobia/phonophobia	(–)

*F, female; GEN, gaze-evoked nystagmus*.

### Genetic Analysis

An average of 5.49 billion bases were generated per individual, with an average sequencing depth of approximately 63 in the target region, achieving the high quality of the sequencing (Supplementary Table 1). An initial screening of genes causing FHM and EA revealed no causative mutation in *CACNA1A, ATP1A2, SCN1A, KCNA1, CACNB4*, or *SLC1A3*.

The number of SNVs and indels identified per individual ranged from 120,657 to 123,359. Among them, a total of 2,586 variants were shared by all three individuals, and 508 of them were heterozygous non-synonymous missense variants, stop codons, coding indels, and splice-site variants at exon-intron boundaries (Figure 2). Next, 36 rare variants were filtered after excluding common variants with a MAF of >0.001 in public databases including gnomAD and KRGDB (Supplementary Table 2), but only two candidate variants in *CDC27* and *TRPM7* were prioritized by the functional annotations (Table 2). Both genes were found to be widely expressed in the brain including the cerebellum in public databases such as GTEx, BioGPS, and SAGE (Supplementary Figure 2).

**Table 2:** Candidate variants identified using bioinformatics tools after applying filtering and prioritization processes.

**Chr**	**Position**	**Gene**	**cDNA**	**Protein**	**Variant effect**	**PAVAR score**	**CADD**	**FATHMM**	**ACMG classification**	**MAF**
15	50885896	*TRPM7*	c.3526C>T	p.Gln1176*	Nonsense	7	38	(–)	Pathogenic (PVS1,PS3,PM2,PP1,PP3,PP4)	NR
17	45258960	*CDC27*	c.71C>G	p.Ala24Gly	Missense	6	32	−3.23	Uncertain significance (PM2,PP1,PP3,PP4)	NR

Transcript ID: TRPM7, NM_017672.5; CDC27, NM_001114091.2.

Evidence code descriptions according to the ACMG classification: PM, pathogenic moderate; PP, pathogenic supporting; PS, pathogenic strong; PVS, pathogenic very strong.

ACMG, American College of Medical Genetics and Genomics; CADD, Combined Annotation-Dependent Depletion; Chr, chromosome, FATHMM, Functional Analysis Through Hidden Markov Models; MAF, minor allele frequency; NR, not reported; PAVAR, pathogenic variants risk composite score.

The *CDC27* variant was a missense mutation c.71C>G of exon 2, which results in the amino acid substitution of alanine by glycine at codon 24. This gene participates in regulation of the cell cycle by encoding the anaphase-promoting complex, and is known to be associated with breast and prostate cancers. However, this variant was classified as “uncertain significance” in ACMG guidelines based on only one moderate (PM2) and three supporting (PP1, PP3, PP4) pathogenic criterion. Furthermore, a literature review did not reveal any evidence for a relationship between the *CDC27* and our family's phenotypes including migraine, vertigo, vestibular or ataxic disorders. Thus, this variant was excluded as a candidate gene for VM.

**Figure 3:**
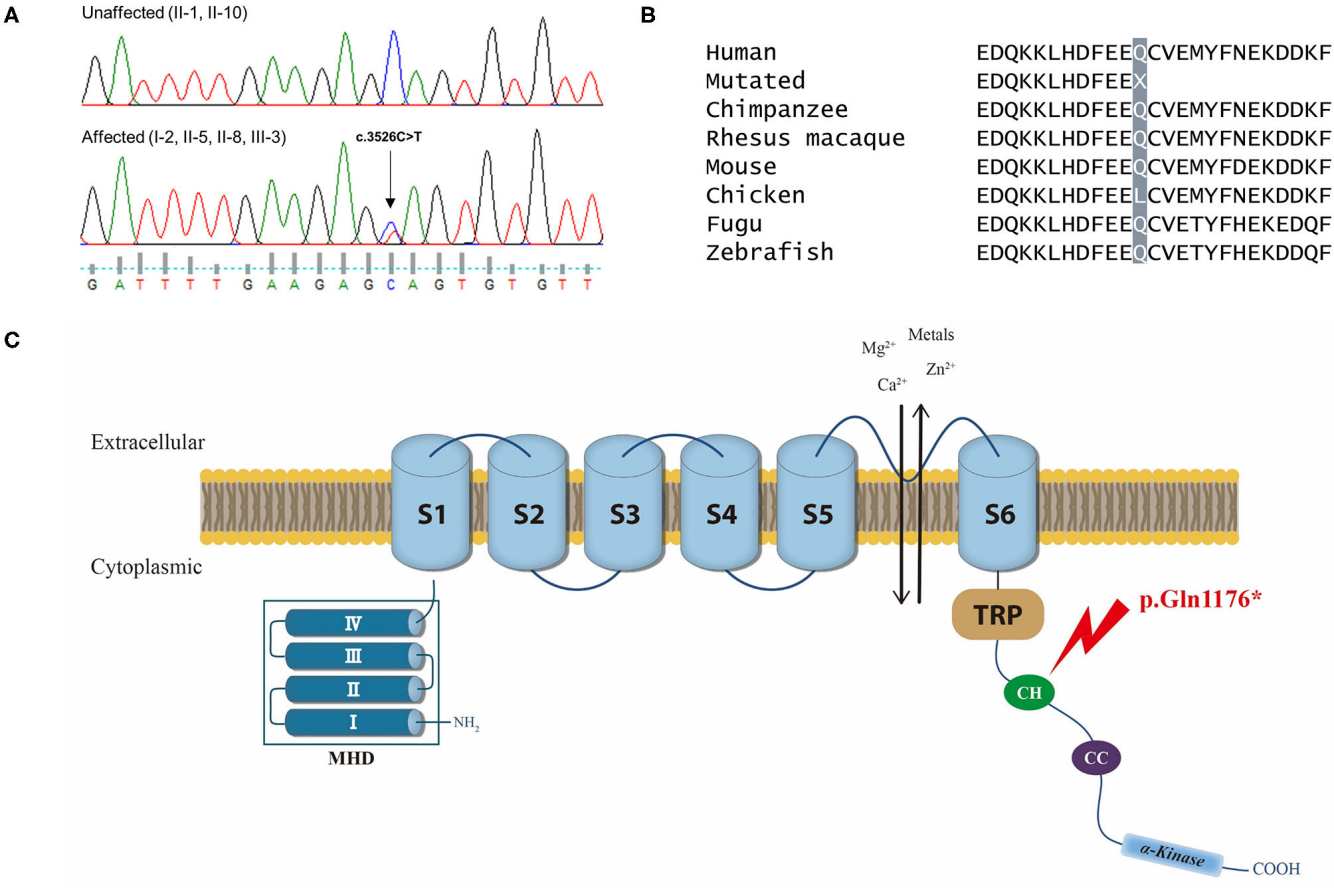
**(A)** Chromatograms of the affected individuals showing a heterozygous nonsense mutation (c.3526C>T) in exon 25 of *TRPM7*, which would result in a premature stop codon (p.Gln1176*). **(B)** Conservation of the mutated residue (Gln, Q) of a “connecting helix” (CH) in the C-terminus, highlighted in gray. **(C)** Schematic representation of the TRPM7 channel and localization of the mutation. The TRPM7 channel comprises a tetrameric complex with each subunit containing six-transmembrane segments (S1–S6) and a pore-forming loop between S5 and S6. Its cytoplasmic N-terminus has four regions of TRPM subfamily homology domain (MHD), whereas the C-terminus contains a TRP box of highly conserved residues, a coiled-coil (CC) domain, and an atypical serine/threonine protein kinase domain. The mutation (p.Gln1176*) is located in a CH that links the transmembrane segment and the CC domain in the C-terminus (arrow).

The *TRPM7* variant was a nonsense mutation c.3526C>T of exon 25 that cause a premature stop codon and loss-of-function in the protein. This variant was also present in another affected individual (I-2), while it was absent in the unaffected individuals (II-1 and II-10), 100 normal controls, gnomAD and KRGDB (Figures 3A,B). By applying ACMG guidelines, the variant was considered “pathogenic” based on one very strong (PVC1), one strong (PS3), one moderate (PM2), and three supporting (PP1, PP3, PP4) pathogenic criterion. The TRPM7 channel belongs to the melastatin subfamily of TRP channels, and it plays a crucial role in maintaining intracellular Ca^2+^ and Mg^2+^ homeostasis (Figure 3C). This has led to proposals that genetic variations in *TRPM7* influence the susceptibility to neurodegenerative diseases (15, 16). In addition, SNVs within some TRP genes have been linked to migraine susceptibility (5, 25–27). Therefore, the *TRPM7* variant may be a causal variant for explaining the phenotypes in the present family.

## Discussion

This study identified a nonsense mutation in *TRPM7* in a Korean family with VM. This variant may cause a premature stop codon and loss-of-function in the protein, thus contributing to the development the VM phenotype by altering the homeostasis of intracellular ions.

Several hypotheses have been proposed for explaining the pathophysiology of VM, which remains unclear. The genetic susceptibility of migraine suggests ionic channelopathy involved in glutamate homeostasis as the underlying pathophysiology of VM (28, 29). Indeed, there is accumulating evidence that Ca^2+^ channels could be involved in migraine and VM pathophysiology. Mutations in *CACNA1A*, which encodes Cav2.1, the α1A subunit of the P/Q-type voltage-gated Ca^2+^ channel, cause three neurological channelopathies: FHM type 1, EA type 2 (EA2), and spinocerebellar ataxia type 6 (30). Patients with FHM or EA2 often experience paroxysmal vertigo, and more than half of EA2 patients have migraine that meets the ICHD criteria. Some members of the present family also presented with hemiplegia and dysarthria during their episodes. Nevertheless, previous studies failed to detect mutations in patients with VM in the genes causing FHM or EA, such as *CACNA1A, ATP1A2, SCN1A*, and *CACNB4* (12, 13), which is consistent with the present study.

*TRPM7* reported here encodes the cation selective ion channel that is highly permeable to Ca^2+^, Mg^2+^, and metal ions such as Zn^2+^ (16). This channel comprises a tetrameric complex, with each subunit containing six-transmembrane segments (S1–S6) and a pore-forming loop between S5 and S6 [Figure 3C; (31)]. Its C-terminus contains a TRP box of highly conserved residues, a coiled-coil (CC) domain, and an atypical serine/threonine protein kinase domain (32). The *TRPM7* mutation identified in the present study is located in a long “connecting helix” that links the transmembrane segment and the CC domain in the C-terminus, thus predicting truncation of the C-terminus including the CC and kinase domains (32). These domains play an important role in the tetrameric assembly of the channel or in regulating channel function by Mg^2+^ nucleotides (31–34). Therefore, the mutation identified in the present study seems to affect TRPM7 channel activity via structural instability or functional impairment. This hypothesis is supported by a previous animal study which generated knockout mice with the deletion of TRPM7 kinase domain (35). These mice carried truncated mutation quite similar to the mutation described in this study. Although homozygous knockout mice caused embryonic lethality, heterozygous mice were viable, but showed abnormality in the regulation of Mg^2+^ homeostasis and alterations in TRPM7 channel properties. These mice exhibited abnormal behaviors such as clasping, tremor, seizure, and violent sudden leaps as a reaction to the light and noise, similar to photophobia and phonophobia seen in VM. Furthermore, one of the affected individuals in this family had a history of recurrent seizure.

Ca^2+^ and Mg^2+^ play important roles in regulating various neuronal functions. In particular, they exert opposite effects in the signaling of the excitatory neurotransmitter glutamate: glutamate release is triggered by an influx of Ca^2+^, whereas Mg^2+^ inhibits glutamate release by antagonizing Ca^2+^ (36). Thus, a tight balance between Ca^2+^ and Mg^2+^ is needed in order to maintain the proper excitability of neurons. Since TRPM7 channels that are abundantly expressed in neuronal cells are highly selective to Ca^2+^ and Mg^2+^ (37, 38), mutation in *TRPM7* may cause alterations in Ca^2+^ and Mg^2+^ homeostasis and neuronal excitability. Migrainous headache is the consequence of cortical spreading depression evoked by glutamate release and N-methyl-D-aspartate (NMDA) receptor activation in the brain. These processes have been linked to low Mg^2+^ concentrations, which contribute to the hyperexcitability of the NMDA receptor (36). A beneficial effect of Mg^2+^ supplementation has also been reported in migraine patients (39). In addition, low Mg^2+^ concentrations increase the amount of substance P released, which is a neuroinflammatory mediator (40). Since the TRPM7 channel is an important Mg^2+^ transporter, it may contribute to VM attacks by affecting intracellular Ca^2+^ and Mg^2+^ homeostasis.

Recently, several TRP channels have been linked to migraine pathophysiology, including TRPV1, TRPV4, TRPM8, and TRPA1 (19). These channels are expressed on trigeminal sensory neurons that innervate the meninges (20, 41). The activation of TRP channels promotes the excitation of nociceptive afferent fibers and potentially leads to pain and allodynia (19, 20). In addition, it can elicit the release of CGRP, causing vasodilation and neurogenic inflammation (19, 42, 43). Several SNPs in *TRPV1, TRPV3*, and *TRPM8* were found to be associated with migraine susceptibility in meta-analyses of observational studies and GWAS (5, 25–27). Based on these findings, TRP channels have been proposed as a therapeutic target in migraine (19, 21, 22, 44). More studies are needed to better explore the potential role for these channels including *TRPM7* in migraine pathophysiology.

This study was subjective to some potential limitations. We did not perform functional study determining pathogenicity of the candidate variants. Despite the rarity and putative pathogenicity in functional annotations, establishing the pathogenicity of the variants may be difficult without a functional assay. However, previous functional studies demonstrated that the deletion of TRPM7 kinase domain reduced channel activity and increased its sensitivity to Mg^2+^ inhibition (33–35). Another limitation is difficulty in detecting copy number variations (CNVs) through whole-exome sequencing. CNV analysis can aid the detection of large deletions or duplications of causative genes, but whole-genome sequencing may be more suitable than whole-exome sequencing for CNV analysis. Finally, we used the hg19 as the reference genome for the reads mapping instead of hg38.

In summary, we have presented the clinical characteristics of a Korean family with VM. Whole-exome sequencing identified a potential disease-causing variant in *TRPM7*, which may cause alterations in intracellular Ca^2+^ and Mg^2+^ homeostasis and neuronal excitability. Our results highlight *TRPM7* as a novel candidate gene for VM.

## Data Availability Statement

The raw data supporting the conclusions of this article will be made available by the authors, without undue reservation.

## Ethics Statement

The studies involving human participants were reviewed and approved by Pusan National University Yangsan Hospital. The patients/participants provided their written informed consent to participate in this study.

## Author Contributions

EO conducted the experiments and interpretation of the data, and wrote the manuscript. J-HS, JC, S-YC, and K-DC contributed to the interpretation and analysis of data. J-HC conducted the design and conceptualization of the study, interpretaion of the data, and revised the manuscript. All authors contributed to the article and approved the submitted version.

## Conflict of Interest

The authors declare that the research was conducted in the absence of any commercial or financial relationships that could be construed as a potential conflict of interest.

## References

[1] Lempert T Olesen J Furman J Waterston J Seemungal B Carey J. Vestibular migraine: diagnostic criteria. J Vestib Res. (2012) 22:167–72. 10.3233/VES-2012-0453 23142830

[2] Headache Classification Committee of the International Headache Society (IHS) The International classification of headache disorders, 3rd Edition. Cephalalgia. (2018) 38:1–211. 10.1177/0333102417738202 29368949

[3] Frejo L Giegling I Teggi R Lopez-Escamez JA Rujescu D. Genetics of vestibular disorders: pathophysiological insights. J Neurol. (2018) 263(Suppl. 1):S45–53. 10.1007/s00415-015-7988-9 27083884 PMC4833787

[4] Roman-Naranjo P Gallego-Martinez A Lopez Escamez JA. Genetics of vestibular syndromes. Curr Opin Neurol. (2018) 31:105–10. 10.1097/WCO.0000000000000519 29095749

[5] Chasman DI Schürks M Anttila V Vries BD Schminke U Launer LJ. Genome-wide association study reveals three susceptibility loci for common migraine in the general population. Nat Genet. (2011) 43:695–8. 10.1038/ng.856 21666692 PMC3125402

[6] de Boer I van den Maagdenberg AMJM Terwindt GM. Advance in genetics of migraine. Curr Opin Neurol. (2019) 32:413–21. 10.1097/WCO.0000000000000687 30883436 PMC6522206

[7] Jen JC Familial Hemiplegic Migraine. GeneReviews. Seattle, WA: University of Washington (2001).

[8] Bahmad F Jr DePalma SR Merchant SN Bezerra RL Oliveira CA Seidman CE. Locus for familial migrainous vertigo disease maps to chromosome 5q35. Ann Otol Rhinol Laryngol. (2009) 118:670–6. 10.1177/000348940911800912 19810609 PMC2767209

[9] Peddareddygari LR Kramer PD Hanna PA Levenstien MA Grewal RP. Genetic analysis of a large family with migraine, vertigo, and motion sickness. Can J Neurol Sci. (2019) 46:512–7. 10.1017/cjn.2019.64 31258098

[10] Lee H Jen JC Cha YH Nelson SF Baloh RW. Phenotypic and genetic analysis of a large family with migraine-associated vertigo. Headache. (2008) 48:1460–7. 10.1111/j.1526-4610.2007.01002.x 18081823 PMC2846425

[11] Lee H Jen JC Wang H Chen Z Mamsa H Sabatti C. A genome-wide linkage scan of familial benign recurrent vertigo: linkage to 22q12 with evidence of heterogeneity. Hum Mol Genet. (2006) 15:251–8. 10.1093/hmg/ddi441 16330481

[12] Kim JS Yue Q Jen JC Nelson SF Baloh RW. Familial migraine with vertigo: no mutations found in CACNA1A. Am J Med Genet. (1998) 79:148–51. 10.1002/(SICI)1096-8628(19980901)79:2<148::AID-AJMG11>3.0.CO;2-J 9741473

[13] von Brevern M Ta N Shankar A Wiste A Siegel Anne Radtke A. Migrainous vertigo: mutation analysis of the candidate genes CACNA1A, ATP1A2, SCN1A, and CACNB4. Headache. (2006) 46:1136–41. 10.1111/j.1526-4610.2006.00504.x 16866717

[14] Clapham DE. TRP channels as cellular sensors. Nature. (2003) 426:517–24. 10.1038/nature02196 14654832

[15] Venkatachalam K Montell C. TRP channels. Annu Rev Biochem. (2007) 76:387–417. 10.1146/annurev.biochem.75.103004.142819 17579562 PMC4196875

[16] Sun Y Sukumaran P Schaar A Singh BB. TRPM7 and its role in neurodegenerative diseases. Channels. (2015) 9:253–61. 10.1080/19336950.2015.1075675 26218331 PMC4826135

[17] Markó L Mannaa M Haschler TN Krämer S Gollasch M. Renoprotection: focus on TRPV1, TRPV4, TRPC6 and TRPM2. Acta Physiol. (2017) 219:589–612. 10.1111/apha.12828 28028935

[18] Tajada S Villalobos C. Calcium permeable channels in cancer hallmarks. Front Pharmacol. (2020) 11:968. 10.3389/fphar.2020.00968 32733237 PMC7358640

[19] Benemei S Dussor G. TRP channels and migraine: recent developments and new therapeutic opportunities. Pharmaceuticals. (2019) 12:54. 10.3390/ph12020054 30970581 PMC6631099

[20] Huang D Li S Dhaka A Story GM Cao YQ. Expression of the transient receptor potential channels TRPV1, TRPA1 and TRPM8 in mouse trigeminal primary afferent neurons innervating the dura. Mol Pain. (2012) 8:66. 10.1186/1744-8069-8-66 22971321 PMC3489865

[21] Dussor G Yan J Xie JY Ossipov MH Dodick DW Porreca F. Targeting TRP channels for novel migraine therapeutics. ACS Chem Neurosci. (2014) 5:1085–1096. 10.1021/cn500083e 25138211 PMC4240253

[22] Benemei S Fusi C Trevisan G Geppetti P. The TRPA1 channel in migraine mechanism and treatment. Br J Pharmacol. (2014) 171:2552–67. 10.1111/bph.12512 24206166 PMC4008999

[23] Requena T Gallego-Martinez A Lopez-Escamez JA. A pipeline combining multiple strategies for prioritizing heterozygous variants for the identification of candidate genes in exome datasets. Hum Genomics. (2017) 11:11. 10.1186/s40246-017-0107-5 28532469 PMC5441048

[24] Richards S Aziz N Bale S Bick D Das S Gastier-Foster J. Standards and guidelines for the interpretation of sequence variants: a joint consensus recommendation of the American college of medical genetics and genomics and the association for molecular Pathology. Genet Med. (2015) 17:405–24. 10.1038/gim.2015.30 25741868 PMC4544753

[25] Carreño O Corominas R Fernández-Morales J Camina M Sobrido MJ Fernandez JM. SNP variants within the vanilloid TRPV1 and TRPV3 receptor genes are associated with migraine in the Spanish population. Am J Med Genet B Neuropsychiatr Genet. (2012) 159B:94–103. 10.1002/ajmg.b.32007 22162417

[26] Key FM Abdul-Aziz MA Mundry R Peter BM Sekar A D'Amato M. Human local adaptation of the TRPM8 cold receptor along a latitudinal cline. PLoS Genet. (2018) 14:e1007298. 10.1371/journal.pgen.1007298 29723195 PMC5933706

[27] Chen SP Fuh JL Chung MY Lin YC Liao YC Wang YF. Genome-wide association study identifies novel susceptibility loci for migraine in Han Chinese resided in Taiwan. Cephalalgia. (2018) 38:466–475. 10.1177/0333102417695105 28952330

[28] Albury CL Stuart S Haupt LM Griffiths LR. Ion channelopathies and migraine pathogenesis. Mol Genet Genomics. (2017) 292:729–39. 10.1007/s00438-017-1317-1 28389699

[29] Murofushi T Tsubota M Kitao K Yoshimura E. Simultaneous presentation of definite vestibular migraine and definite Ménière's disease: overlapping syndrome of two diseases. Front Neurol. (2018) 9:749. 10.3389/fneur.2018.00749 30250448 PMC6139324

[30] Choi KD Choi JH. Episodic ataxias: clinical and genetic features. J Mov Disord. (2016) 9:129–135. 10.14802/jmd.16028 27667184 PMC5035943

[31] Park HS Hong C Kim BJ So I. The pathophysiologic roles of TRPM7 channel. Korean J Physiol Pharmacol. (2014) 18:15–23. 10.4196/kjpp.2014.18.1.15 24634592 PMC3951819

[32] Duan J Li Z Li J Hulse RE Santa-Cruz A Valinsky WC. Structure of the mammalian TRPM7, a magnesium channel required during embryonic development. Proc Natl Acad Sci USA. (2018) 115:E8201–10. 10.1073/pnas.1810719115 30108148 PMC6126765

[33] Runnels LW Yue L Clapham DE. TRP-PLIK, a bifunctional protein with kinase and ion channel activities. Science. (2001) 291:1043–7. 10.1126/science.1058519 11161216

[34] Demeuse P Penner R Fleig A. TRPM7 channel is regulated by magnesium nucleotides via its kinase domain. J Gen Physiol. (2006) 127:421–34. 10.1085/jgp.200509410 16533898 PMC2151514

[35] Ryazanova LV Rondon LJ Zierler S Hu Z Galli J Yamaguchi TP. TRPM7 is essential for Mg(2+) homeostasis in mammals. Nat Commun. (2010) 1:109. 10.1038/ncomms1108 21045827 PMC3060619

[36] de Baaij JH Hoenderop JG Bindels RJ. Magnesium in man: implications for health and disease. Physiol Rev. (2015) 95:1–46. 10.1152/physrev.00012.2014 25540137

[37] Kunert-Keil C Bisping F Krüger J Brinkmeier H. Tissue-specific expression of TRP channel genes in the mouse and its variation in three different mouse strains. BMC Genomics. (2006) 7:159. 10.1186/1471-2164-7-159 16787531 PMC1557673

[38] Fonfria E Murdock PR Cusdin FS Benham CD Kelsell RE McNulty S. Tissue distribution profiles of the human TRPM cation channel family. J Recept Signal Transduct Res. (2006) 26:159–78. 10.1080/10799890600637506 16777713

[39] Dolati S Rikhtegar R Mehdizadeh A Yousefi M. The role of magnesium in pathophysiology and migraine treatment. Biol Trace Elem Res. (2020) 196:375–83. 10.1007/s12011-019-01931-z 31691193

[40] Weglicki WB Phillips TM. Pathobiology of magnesium deficiency: a cytokine/neurogenic inflammation hypothesis. Am J Physiol. (1992) 263(3 Pt 2):R734–7. 10.1152/ajpregu.1992.263.3.R734 1384353

[41] Burgos-Vega C Moy J Dussor G. Meningeal afferent signaling and the pathophysiology of migraine. Prog Mol Biol Transl Sci. (2015) 131:537–64. 10.1016/bs.pmbts.2015.01.001 25744685

[42] Veldhuis NA Poole DP Grace M McIntyre P Bunnett NW. The G protein-coupled receptor-transient receptor potential channel axis: molecular insights for targeting disorders of sensation and inflammation. Pharmacol Rev. (2015) 67:36–73. 10.1124/pr.114.009555 25361914

[43] Russell FA King R Smillie SJ Kodji X Brain SD. Calcitonin gene-related peptide: physiology and pathophysiology. Physiol Rev. (2014) 94:1099–142. 10.1152/physrev.00034.2013 25287861 PMC4187032

[44] Artero-Morales M González-Rodríguez S Ferrer-Montiel A. TRP channels as potential targets for sex-related differences in migraine pain. Front Mol Biosci. (2018) 5:73. 10.3389/fmolb.2018.00073 30155469 PMC6102492

