# Supplementary material for: TRPM7 as a Candidate Gene for Vestibular Migraine
Source: Front Neurol. 2020 Oct 23;11:595042. doi: 10.3389/fneur.2020.595042 (PMC7649787; doi:10.3389/fneur.2020.595042)
Supplement: Supplementary file 1 [file Data_Sheet_1.docx]

**Supplementary Table 1.** Quality parameters of next-generation sequencing results for three affected individuals in this study

| Patient ID | Total reads (bam) | Mapping read (%) | On target read (%) | Total yield (Gbp) | Average depth | % Covered (> x 20) |
| --- | --- | --- | --- | --- | --- | --- |
| II-5 | 47,760,312 | 44,543,754 (99.32) | 36,978,988 (82.45) | 4.82 | 62.47 | 93.87 |
| II-8 | 57,522,266 | 53,402,914 (99.07) | 41,423,392 (76.84) | 5.81 | 64.94 | 93.55 |
| III-3 | 57,824,094 | 54,217,486 (98.94) | 41,059,111 (74.93) | 5.84 | 64.12 | 93.60 |

**Supplementary Table 2.** Prioritized rare SNVs found in three affected individuals

| Chr | Position | Gene | Ref Sequence | cDNA | Protein | Variant effect | PAVAR score | CADD | FATHMM | dbSNP | MAF |
| --- | --- | --- | --- | --- | --- | --- | --- | --- | --- | --- | --- |
| 1 | 155874197 | *RIT1* | NM_001256821.1 | c.385C>T | p.Arg129Cys | Missense | 6 | 25.8 | -1.15 | rs375724784 | 1.77x10^-5^ |
| 1 | 171236733 | *FMO1* | NM_001282692.1 | c.196A>T | p.Ser66Cys | Missense | 7 | 26.2 | 0.46 | (-) | NR |
| 1 | 6530940 | *PLEKHG5* | NM_001265592.1 | c.1634C>T | p.Ala545Val | Missense | 5 | 25.0 | -0.32 | rs199839017 | 7.89x10^-5^ |
| 1 | 154923846 | *PBXIP1* | NM_020524.2 | c.271G>C | p.Val91Leu | Missense | 0 | 0.0 | 2.63 | rs759513344 | 7.96x10^-5^ |
| 1 | 154995707 | *DCST2* | NM_144622.2 | c.1991A>G | p.Gln664Arg | Missense | 1 | 0.2 | 2.00 | rs763399486 | 9.63x10^-5^ |
| 2 | 179474874 | *TTN* | NM_001267550.2 | c.51379G>C | p.Val17127Leu | Missense | 4 | 17.1 | 0.35 | rs397517603 | 4.03x10^-6^ |
| 3 | 44373355 | *TOPAZ1* | NM_001145030.1 | c.4931G>A | p.Arg1644His | Missense | 5 | 22.6 | 2.61 | rs1399322081 | 3.80x10^-5^ |
| 5 | 118965483 | *FAM170A* | NM_182761.3 | c.20G>A | p.Arg7Lys | Missense | 5 | 25.0 | 1.29 | rs770200505 | 1.20x10^-5^ |
| 5 | 121726840 | *SNCAIP* | NM_001308100.1 | c.11C>T | p.Pro4Leu | Missense | 6 | 27.4 | 2.29 | rs1397703397 | NR |
| 5 | 176318106 | *HK3* | NM_002115.2 | c.346C>T | p.His116Tyr | Missense | 2 | 19.8 | -5.24 | rs77506590 | 1.19x10^-5^ |
| 7 | 29923915 | *WIPF3* | NM_001080529.2 | c.805C>T | p.Pro269Ser | Missense | 4 | 22.1 | 0.91 | (-) | NR |
| 7 | 66240266 | *RABGEF1* | NM_014504.2 | c.232G>A | p.Ala78Thr | Missense | 4 | 22.1 | 0.91 | (-) | NR |
| 7 | 139719884 | *TBXAS1* | NM_001166253.1 | c.1728G>C | p.Lys576Asn | Missense | 4 | 24.3 | -0.44 | (-) | 3.98x10^-6^ |
| 7 | 149488468 | *SSPO* | NM_198455.2 | c.5000T>C | p.Ile1667Thr | Missense | 0 | 11.0 | 0.51 | rs760661789 | 1.68x10^-5^ |
| 9 | 130536485 | *SH2D3C* | NM_170600.2 | c.299C>T | p.Ala100Val | Missense | 0 | 0.0 | 2.78 | rs372890268 | 8.02x10^-5^ |
| 10 | 115905415 | *CCDC186* | NM_018017.2 | c.994C>G | p.Leu332Val | Missense | 4 | 24.2 | 1.17 | rs755942104 | NR |
| 10 | 134259261 | *C10orf91* | NM_173541.2 | c.91G>C | p.Asp31His | Missense | 1 | 1.1 | 0.04 | (-) | NR |
| 10 | 123724872 | *NSMCE4A* | NM_017615.2 | c.682G>A | p.Val228Met | Missense | 3 | 18.5 | 0.80 | rs746126284 | 7.78x10^-5^ |
| 11 | 17591891 | *OTOG* | NM_001277269.1 | c.1945G>A | p.Val649Met | Missense | 5 | 25.0 | 0.25 | (-) | NR |
| 12 | 6483993 | *SCNN1A* | NM_001159576.1 | c.134T>C | p.Leu45Pro | Missense | 1 | 12.4 | -1.02 | (-) | 1.19x10^-5^ |
| 12 | 113545930 | *RASAL1* | NM_001193520.1 | c.1475C>T | p.Ala492Val | Missense | 5 | 25.5 | 2.39 | rs772684767 | 4.61x10^-5^ |
| 13 | 52544777 | *ATP7B* | NM_000053.3 | c.1394G>T | p.Arg465Met | Missense | 1 | 1.2 | -3.88 | (-) | NR |
| 13 | 44411511 | *CCDC122* | NM_144974.3 | c.727A>C | p.Lys243Gln | Missense | 5 | 25.7 | 1.55 | rs746094814 | 1.14x10^-4^ |
| 13 | 114322113 | *GRK1* | NM_002929.2 | c.412C>A | p.Pro138Thr | Missense | 0 | 0.5 | 4.64 | rs753003410 | 1.83x10^-5^ |
| 14 | 45521650 | *FAM179B* | NM_001308120.1 | c.4325G>A | p.Arg1442Gln | Missense | 6 | 29.7 | -0.03 | (-) | 1.60x10^-5^ |
| 14 | 50788204 | *ATP5S* | NM_001003803.2 | c.44A>G | p.Gln15Arg | Missense | 2 | 17.2 | 1.51 | (-) | NR |
| 15 | 50885896 | *TRPM7* | NM_017672.5 | c.3526C>T | p.Gln1176* | Nonsense | 7 | 38.0 | (-) | (-) | NR |
| 15 | 59175934 | *SLTM* | NM_024755.2 | c.2887G>A | p.Gly963Arg | Missense | 7 | 31.0 | 2.32 | rs374779296 | 1.19x10^-5^ |
| 15 | 90226853 | *PEX11A* | NM_003847.2 | c.499T>G | p.Phe167Val | Missense | 2 | 6.4 | 1.04 | (-) | 8.00x10^-6^ |
| 15 | 90771582 | *SEMA4B* | NM_020210.3 | c.2221C>T | p.Arg741Trp | Missense | 7 | 29.5 | -1.26 | (-) | 2.61x10^-5^ |
| 15 | 86125041 | *AKAP13* | NM_006738.5 | c.3742A>G | p.Ile1248Val | Missense | 3 | 16.7 | 2.48 | rs114556336 | 2.05x10^-4^ |
| 16 | 3633383 | *SLX4* | NM_032444.2 | c.4868C>T | p.Ala1623Val | Missense | 0 | 1.0 | 5.16 | rs200178217 | 8.15x10^-5^ |
| 16 | 4933587 | *PPL* | NM_002705.4 | c.5069G>A | p.Ser1690Asn | Missense | 4 | 20.9 | -0.82 | (-) | 7.96x10^-5^ |
| 16 | 5058530 | *SEC14L5* | NM_014692.1 | c.1681G>A | p.Gly561Ser | Missense | 0 | 9.8 | -0.38 | rs748766480 | 5.33x10^-5^ |
| 17 | 45258960 | *CDC27* | NM_001114091.2 | c.71C>G | p.Ala24Gly | Missense | 6 | 32.0 | -3.23 | (-) | NR |
| 22 | 26231363 | *MYO18B* | NM_032608.5 | c.3161T>C | p.Leu1054Pro | Missense | 6 | 24.1 | -0.86 | rs765870937 | 7.13x10^-5^ |

*CADD* Combined Annotation-Dependent Depletion, *Chr* chromosome, *FATHMM* Functional Analysis Through Hidden Markov Models, *MAF* minor allele frequency, *NR* not reported, *PAVAR* pathogenic variants risk composite score

**Supplementary Figure 1.** Air-conduction audiograms showing normal hearing functions in the affected individuals with auditory symptoms


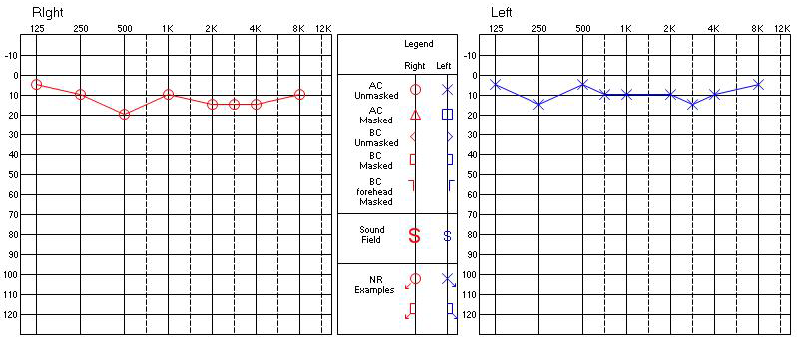
**II-5**

**II-8**


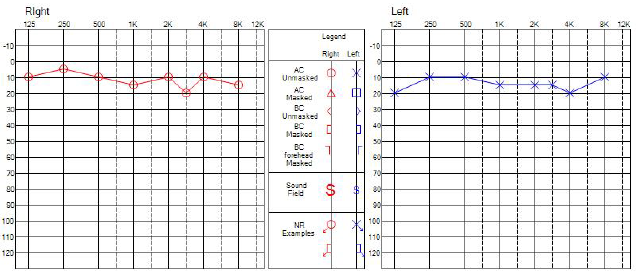


**Supplementary Figure 2.** The gene expression patterns of *TRPM7* (A) and *CDC27* (B)


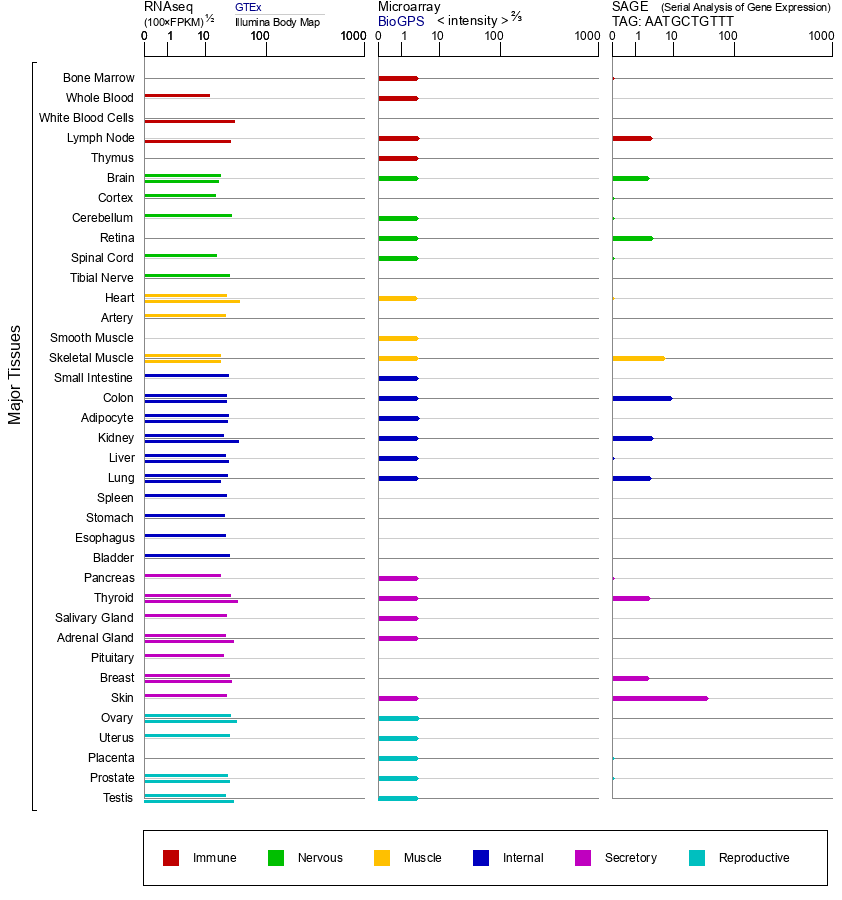

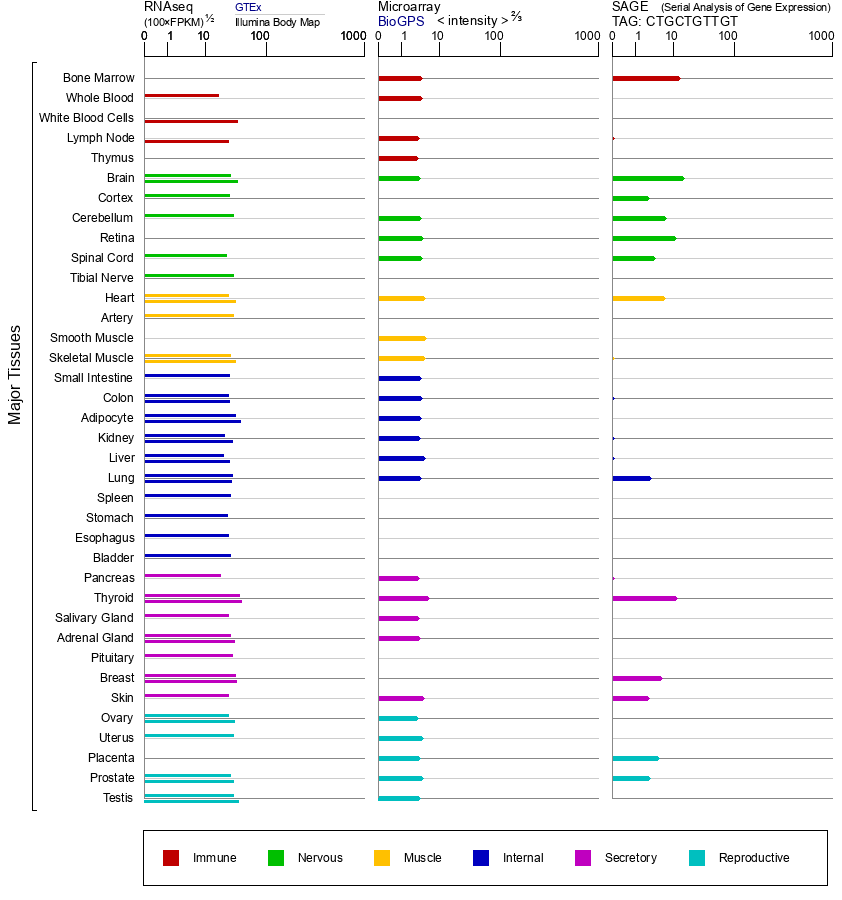


**A**

**B**
